# Recent Innovations in Collagen Corneal Cross-linking; a Mini Review

**DOI:** 10.2174/1874364101711010217

**Published:** 2017-07-31

**Authors:** Iraklis Vastardis, Brigitte Pajic-Eggspuehler, Charis Nichorlis, Jörg Mueller, Bojan Pajic

**Affiliations:** 1 Swiss Eye Research Foundation, Eye Clinic ORASIS, Titlisstrasse 44, 5734 Reinach, Switzerland; 2 University of Novi Sad, Faculty of Physics, Novi Sad, Serbia; 3 Medical faculty, Military Medical Academy, University of defence Belgrade, Serbia

**Keywords:** Corneal Cross Linking, Riboflavin, Higher fluence, Keratoconus, Dresden Protocol, Biomachanics

## Abstract

**Background:**

The introduction of corneal cross-linking (CXL) with ultraviolet-A (UVA) and Riboflavin photosensitizer (Vit B_2_) from Seiler *et al.*, revolutionized the treatment of Keratoconus and other corneal ectatic diseases.

Today, the commonly known epithelium off Dresden protocol is in clinical use for the last 15 years with great success and regarded by many as the golden standard.

**Methods:**

With several studies demonstrating its simplicity, efficacy and safety this revolutionary method, paved the way for new therapies and strategies in the treatment of corneal ectatic diseases and changed our understanding in corneal biomechanics. Recent scientific and technological advances enabled the creation of various modifications of the initial CXL protocol and the formation of new ones.

**Conclusion:**

This work highlights the recent advances of CXL, such as the role of oxygen, higher fluence and shorter irradiation times as well as the various clinical applications and updates of this method.

## 1 INTRODUCTION

Corneal cross-linking (CXL) was introduced in the late 1990s by Seiler *et al.* [1, 2]. Until that time the conservative therapeutic approach of Keratoconus and other corneal ectatic diseases, involved mostly the fitting of hard contact lenses as an improvement of corrected visual acuity and as a mean to halt the progression through corneal adaptation on the posterior surface of the contact lens [3, 4]. In most cases, the treatment of choice after an extended visual loss over time resulted in a penetrating Keratoplasty [5].

In normal conditions, physiological enzymatic collagen cross linking by lysyl oxidase creates stability in the corneal stroma, whereas decreased lysyl oxidase activity contribute to its instability [6].

Being known that natural cross linking could occur in patients with diabetes and that Keratoconus is almost absent in such patients gave birth to the idea that induction of cross links in cornea tissue could result in hardening and stiffening of the cornea [1, 2, 7].

Today, the commonly known epithelium off Dresden protocol is in clinical use for the last 15 years with great success and regarded from many as the golden standard.

## 2 DRESDEN PROTOCOL: CORNEAL CROSS LINKING, THE EPI-off METHOD

In order to perform a cross linking reaction to the cornea according to the Dresden Protocol, we need a inducer or light emitting source, in this case UVA light, a photosensitizer, Riboflavin Vitamin B_2_ and as recently proposed, abundant oxygen [2, 8, 9]. In the Dresden protocol described first by Wollensack *et al.* [2], the cornea is anesthetized then the epithelium is removed from 7 to 9 mm in diameter and the corneal stroma is saturated with Riboflavin for 30 minutes. After the first 30 minutes, the irradiation of the cornea for another 30 minutes begins with UVA light of 3 mW/cm^2^ for a total of UVA fluence of 5.4 J/cm^2^. During this time additional Riboflavin is instilled to the corneal stroma every 5 minutes.

The results in terms of efficacy and safety in treatment of Keratoconus and corneal ectatic diseases with the use of CXL are well known and documented [1, 2, 6, 8-17]. Regarding long term safety and efficacy O`Brart *et al.* showed improvements in Keratoconus parameters at five to seven years follow up and Raiskup *et al.* showed evidence of CXL being safe and effective in treating progressive Keratoconus by achieving long term stabilization in their ten years follow up results [13, 14].

However, while over 1000 papers on the standard Dresden Epi-off protocol nowadays exist, most of them are non-randomized case series or retrospective studies. Recently, a number of randomized controlled trials have been conducted. Wittig-Silva *et al.* published their three years results of a prospective, randomized controlled trial of 94 eyes, 48 in the control group and 46 in the CXL treatment group and found a sustained improvement in topographic Keratoconus parameters and BCVA after CXL while the control group showed further progression [15].

A wide-ranging review by Sykakis *et al.* for the Cochrane Collaboration analyzed data from 219 eyes drawn from three randomized controlled trials of CXL and concluded that the evidence for the use of CXL in the management of Keratoconus is limited due to the lack of properly conducted randomized controlled trials [16]. Also a meta-analysis by Craig *et al.* included in their analysis only 49 out of 3400 papers found in the literature. They stated that 39/49 studies were graded as very low quality evidence. While statistically significant improvements were found in all efficacy outcomes at 12 months after CXL, they concluded that the remaining uncertainty is duration of benefit in order to establish the procedure's potential benefit in avoiding or delaying disease progression and possibly reducing the need for corneal transplantation [17].

## 3 ADVANCES

As we described above, the epithelium off CXL is in broader use in modern ophthalmology. Recent scientific and technological advances enabled the creation of various modifications of the initial epithelium off CXL protocol that seems to provide equal good results in terms of safety, efficacy and clinical application. This work describes the major innovations of the past decade. With the advance of science in various fields, the key ingredients like Riboflavin, fluence and treatment time were also upgraded and finally from many adopted.

The aim was to create same results of safety and efficacy in the clinical application of CXL in less time and with less patient discomfort.

## 3.1 Riboflavin

Riboflavin is a key component in the process of CXL. As described in the first studies of CXL, a 0,1% Riboflavin 5 phosphate with 20% of dextran T-500 solution was used [2, 6, 11, 12]. Dextran creates a coating of Riboflavin on the ocular surface during the loading phase and acts as a protecting shield by absorbing a great deal of UVA light, reducing in this way the dose of UVA light in total but also the depth of the cross linking effect in the corneal stroma [10-12]. However, the oncotic effect of Dextran Riboflavin results in corneal thinning [18]. According to a study, the practice of closed eyelids during the saturation of the cornea with Riboflavin by removing the speculum in one group of patients and by not removing it in the second group showed a statistically significant difference in central corneal thickness (CCT) reduction between the 2 groups tested (P<.001), with a mean CCT decrease of 62 μm±53 in Group A and 11 ± 35 μm in Group B. Six months after CXL, no statistically significant between-group difference was found in the visual acuity, refraction, keratometry, pachymetry, or endothelium [18, 19]. Furthermore new dextran free Riboflavin 5 phosphate solutions were also introduced recently targeting the oncotic effects [20]. The comparison between standard 20% Dextran Riboflavin and a new hydroxypropyl methylcellulose (HPMC) solution showed a 20% less corneal thinning favoring the new solution [20] and a 10% corneal swelling with the use of hypotonic saline solution [21]. Other studies showed also similar results with use of dextran free Riboflavin solution [22, 23]. Jain *et al.* monitored corneal pachymetry changes during accelerated collagen cross-linking (CXL) for Keratoconus while using isotonic Riboflavin with HPMC and showed no significant decline in the corneal thickness [23].

The use of hypo-osmolar Riboflavin came to broad use in thin corneas after Hafezi *et al.* modified the standard treatment protocol by preoperatively swelling thin corneas to a stromal thickness of at least 400 micron using hypo-osmolar Riboflavin solution. This treatment protocol was performed in a case series of 20 patients, and no complications were observed. Preoperative swelling of the cornea safely broadened the spectrum of CXL indications to thin corneas that would otherwise not be eligible for treatment with the “Epi-Off” protocol [24, 25].

## 4 TRANSEPITHELIAL CORNEAL CROSS LINKING, THE EPI-ON METHOD

The transepithelial CXL or “Epi-on” CXL was introduced recently as a mean of improving comfort for the patient and safety of the procedure by minimizing infections when the epithelium barrier was left in place [26, 27]. Although this method seemed promising there have been mixed results regarding its efficacy and ability to deliver significant amount of Riboflavin in the corneal stroma [28, 29] resulting in some cases in progression of Keratokonus [30, 31]. In order to facilitate better results various ways of improving the epithelium penetration of Riboflavin have been proposed. Various studies suggested that the use of benazalconium Chloride (BAC), ethylendiaminatetetraacetic acid (EDTA) and other chemical agents like tetracaine or pilocarpin could facilitate the diffusion and saturation of Riboflavin in the corneal stroma by loosening the epithelial junctions [26, 27, 32, 33].

Raiskup *et al.* investigated the influence of osmolarity on the transepithelial permeability of Riboflavin solutions in a cross-linking procedure in 36 rabbit corneas using several Riboflavin 0.1% solutions that contained different NaCl and BAC concentrations. He concluded that the transepithelial Riboflavin solution should contain no dextran, but it should include 0.01% BAC and 0.44% NaCl to promote the permeability of Riboflavin through the epithelium, resulting in a sufficient concentration of Riboflavin in the corneal stroma [27]. Other proposed methods include direct introduction of Riboflavin in the corneal stroma through the pocket, needle or ring technique [34-36] and the iontophoresis method [37, 38]. Iontophoresis is a non-invasive method that facilitates the penetration of molecules into tissue by using small amount of electric current [39] and has been in use in ophthalmology mostly for vitreoretinal diseases [40]. Studies have shown that transepithelial collagen cross-linking by iontophoresis might become an effective method for Riboflavin delivery in the corneal stroma, may reduce the duration of the procedure and be more comfortable for the patients. However further long-term studies are necessary to evaluate the efficacy and safety of this modified cross-linking method [38].

## 5 ACCELERATED CORNEAL CROSS LINKING

The UVA light plays a major role and is also a key component for performing CXL. Safety depends on wavelength, irradiance and time of irradiation. Riboflavin absorption peak is regarded most effective at 370 nm, but also was found that at this wavelength adequate protection to the surrounding ocular tissues is provided [10]. In Dresden protocol maximum efficacy of tissue stiffening is provided using 3 mW/cm^2^ of energy for 30 minutes corresponding to fluence of 5.4 J/cm^2^ [2].

The Bunsen-Roscoe law of reciprocity states that a photochemical effect should be similar as long as the total fluence remains constant [41]. Various attempts to accelerate the original protocol resulted in shorter times with higher fluence and the introduction of accelerated Cross Linking [42-47]. Wernli *et al.* investigated the biomechanical strengthening of ex vivo corneal tissue treated with irradiances between 3 mW/cm^2^ and 90 mW/cm^2^ and illumination times from 30 minutes to 1 minute, respectively in a total of 100 porcine eyes and at a constant irradiation dose of 5.4 J/cm^2^ but with different intensities and illumination times randomly assigned into 10 groups [42]. Results showed that the Bunsen-Roscoe reciprocity law is only valid for illumination intensities up to 40 to 50 mW/cm^2^ and illumination times of more than 2 minutes, concluding that further experiments are necessary to validate these results for in vivo human corneal tissue [42].

Kanellopoulos evaluated the safety and efficacy of higher fluence cornea collagen cross linking CXL in twenty-one patients with bilateral keratoconus [43].

After randomization of CXL in one eye (group A) with 7 mw/cm^2^ for 15 minutes and the other eye (group B) with standard 3 mw/cm^2^ for 30 minutes resulted that there was no ectasia progression in any of the cases during the follow-up time studied and no change in the endothelial cell count. Mean follow-up was 46 months. Corneal optical coherence tomography (OCT) revealed diffused light scattering in anterior two-thirds of the cornea stroma, which was more intense and much broader in diameter in group A than in group B [43]. Gatzioufas *et al.* investigated the effect of high-fluence CXL in the management of progressive Keratoconus in a prospective cohort study [44].

CXL was performed as a standard epithelium-off procedure in seven eyes and 7 patients with progressive Keratoconus irradiating with high-fluence at 18 mW/cm^2^ for 5 minutes.

Endothelial cell density (ECD), speed of postoperative epithelial healing, maximal and average keratometric readings (Kmax and Kmean, respectively) of the anterior corneal surface, and corrected distance visual acuity (CDVA) were evaluated preoperatively and at 1 and 6 months after CXL. He concluded that no complications were observed postoperatively suggesting that although the preliminary results are not sufficient for a valid evaluation of the biomechanical effect and the overall safety profile of high-fluence CXL in vivo, they demonstrate that CXL at 18 mW/cm^2^ for 5 minutes affects neither endothelial cell density nor the speed of epithelial healing, an indirect indicator of limbal stem cell function [45]. Regarding the stem cell function Richoz *et al.* concluded that even when using fluence twice as high as the one used in current clinical CXL settings, circumferential UV-A irradiation of the corneal limbus does not alter the regenerative capacity of the limbal epithelial cells, and the expression pattern of the putative stem cell marker p63 remains unchanged suggesting that eccentric CXL may be performed safely in pellucidal marginal degeneration [45]. The efficacy and safety of accelerated CXL is still under investigation [46, 47] and also multicenter clinical trials are currently underway.

## 6 PULSED CORNEAL CROSS LINKING AND THE ROLE OF OXYGEN

Oxygen plays a fundamental role in CXL reaction [9]: Oxygen concentration in the cornea is modulated by UV-A irradiance and temperature and quickly decreased at the beginning of UV-A exposure. The time-dependence of both Type-I and Type-II photochemical mechanisms in corneal cross-linking with Riboflavin are still investigated and needs to be better understood [48] It is suggested that high oxygen availability potentially increases the overall efficacy of Riboflavin UV-A CXL [9]. Hammer *et al.* investigated the biomechanical properties of four groups, 50 each, of porcine corneas at different CXL irradiances [49]. Three groups were exposed to Riboflavin 0.1% and UV-A irradiation of equal total energy 3 mW/cm^2^ for 30 minutes, 9 mW/cm^2^ for 10 minutes, and 18 mW/cm^2^ for 5 minutes. Controls were exposed to Riboflavin 0.1% without irradiation. He concluded that the biomechanical effect of CXL decreased significantly when using high irradiance/short irradiation time settings and that intrastromal oxygen diffusion capacity and increased oxygen consumption is associated with higher irradiances that may be a limiting factor leading to reduced treatment efficiency [49]. Herekar *et al.* first proposed the use of pulsed illumination to increase oxygen concentration during CXL by allowing diffusion of oxygen during pauses [50]. Mazzota *et al.* [51] compared functional results in two cohorts of patients undergoing epithelium-off pulsed (pl-ACXL) and continuous light accelerated corneal collagen crosslinking (cl-ACXL) with dextran-free Riboflavin solution and high-fluence ultraviolet A irradiation. One year after cl-ACXL and pl-ACXL demonstrated Keratoconus stability in both groups. Functional outcomes were found to be better in epithelium-off pulsed light accelerated treatment together with showing a deeper stromal penetration. No endothelial damage was recorded during the follow-up in both groups. This study confirmed that oxygen represents the main driver of collagen crosslinking reaction [51].

 Pulsed light treatment optimized intraoperative oxygen availability thus improving postoperative functional outcomes compared with continuous light treatment. The high oxygen rate in the “epi-off” method could result in more radicals that facilitate cross linking in regard with equivalent dose of continuous UVA [51]. Further comparison studies between pl-ACXL and cl-ACXL in vivo with aid of confocal microscopy and corneal OCT showed epithelial stratification and nerves regeneration improvement in time, being complete at month 6 in both groups without endothelial damage and allowed a precise qualitative analysis of the cornea after epithelium-off PL-ACXL and CL-ACXL treatments [52].

Pulsed CXL seems to be effective in both stiffening the cornea and halting the progression of Keratoconus by increasing the efficiency of high fluence CXL. However more studies needs to surface in order to estimate the irradiance to efficiency ratio in this application of CXL.

## CONCLUSION

The primary goal of corneal collagen cross linking at the time that was incepted and later introduced in ophthalmology was to halt the progression of Keratoconus and of other corneal ectatic diseases. With the advent of new technology and after of more than 10 years of original clinical research, this versatile method, stretched the boundaries in the biomechanical tissue alteration field and extended its clinical application in clinical ophthalmology even more. Nowadays apart from the improvement of the original method with the introduction of new Riboflavin solutions, higher fluence and shorter treatment times, broader use is still discovered in the field of refractive surgery with the advent of Athens [53, 54] and Cretan protocol [55, 56] but also with its application for the treatment of infectious Keratitis and other corneal diseases [57-59].

Corneal cross linking with new indications and treatment protocols on the way clearly remains still a highly versatile and fascinating method.

## References

[1] Spoerl E., Huhle M., Seiler T. (1998). Induction of cross-links in corneal tissue.. Exp. Eye Res..

[2] Wollensak G., Spoerl E., Seiler T. (2003). Riboflavin/ultraviolet-a-induced collagen crosslinking for the treatment of keratoconus.. Am. J. Ophthalmol..

[3] Roth H.W. (1989). Deformation of the central corneal surface following use of hard and soft contact lenses.. Fortschr. Ophthalmol..

[4] Massin M., Denis-Morère A., Ninine G. (1976). Keratoconus and contact lenses (author’s transl).. Klin. Monatsbl. Augenheilkd..

[5] Rabinowitz Y.S. (1998). Keratoconus.. Surv. Ophthalmol..

[6] Raiskup F., Spoerl E. (2013). Corneal crosslinking with riboflavin and ultraviolet A. I. Principles.. Ocul. Surf..

[7] Seiler T., Huhle S., Spoerl E., Kunath H. (2000). Manifest diabetes and keratoconus: a retrospective case-control study.. Graefes Arch. Clin. Exp. Ophthalmol..

[8] Richoz O., Hammer A., Tabibian D., Gatzioufas Z., Hafezi F. (2013). The Biomechanical effect of corneal collagen cross-linking (CXL) with riboflavin and UV-A is oxygen dependent.. Transl. Vis. Sci. Technol..

[9] Kling S., Richoz O., Hammer A., Tabibian D., Jacob S., Agarwal A., Hafezi F. (2015). Increased biomechanical efficacy of corneal cross-linking in thin corneas due to higher oxygen availability.. J. Refract. Surg..

[10] Spoerl E., Mrochen M., Sliney D., Trokel S., Seiler T. (2007). Safety of UVA-riboflavin cross-linking of the cornea.. Cornea.

[11] Caporossi A., Mazzotta C., Baiocchi S., Caporossi T. (2010). Long-term results of riboflavin ultraviolet a corneal collagen cross-linking for keratoconus in Italy: the Siena eye cross study.. Am. J. Ophthalmol..

[12] Raiskup-Wolf F., Hoyer A., Spoerl E., Pillunat L.E. (2008). Collagen crosslinking with riboflavin and ultraviolet-A light in keratoconus: long-term results.. J. Cataract Refract. Surg..

[13] Raiskup F., Theuring A., Pillunat L.E., Spoerl E. (2015). Corneal collagen crosslinking with riboflavin and ultraviolet-A light in progressive keratoconus: ten-year results.. J. Cataract Refract. Surg..

[14] O’Brart D.P., Patel P., Lascaratos G., Wagh V.K., Tam C., Lee J., O’Brart N.A. (2015). Corneal cross-linking to halt the progression of keratoconus and corneal ectasia: even-Year follow-up.. Am. J. Ophthalmol..

[15] Wittig-Silva C., Chan E., Islam F.M., Wu T., Whiting M., Snibson G.R. (2014). A randomized, controlled trial of corneal collagen cross-linking in progressive keratoconus: three-year results.. Ophthalmology.

[16] Sykakis E., Karim R., Evans J.R., Bunce C., Amissah-Arthur K.N., Patwary S., McDonnell P.J., Hamada S. (2015). Corneal collagen cross-linking for treating keratoconus.. Cochrane Database Syst. Rev..

[17] Craig J.A., Mahon J., Yellowlees A., Barata T., Glanville J., Arber M., Mandava L., Powell J., Figueiredo F. (2014). Epithelium-off photochemical corneal collagen cross-linkage using riboflavin and ultraviolet a for keratoconus and keratectasia: a systematic review and meta-analysis.. Ocul. Surf..

[18] Schmidinger G., Pachala M., Prager F. (2013). Pachymetry changes during corneal crosslinking: effect of closed eyelids and hypotonic riboflavin solution.. J. Cataract Refract. Surg..

[19] Soeters N., van Bussel E., van der Valk R., Van der Lelij A., Tahzib N.G. (2014). Effect of the eyelid speculum on pachymetry during corneal collagen crosslinking in keratoconus patients.. J. Cataract Refract. Surg..

[20] Mazzotta C., Baiocchi S., Caporossi T. (2013). Riboflavin 0.1% (VibeX) for the treatment of keratoconus.. Expert Opin. Orphan Drugs.

[21] Sherr E, Kamaev P, Rood-Ojalvo S, Friedman M, Muller D. (2013). Impact of Riboflavin Formulations on Corneal Hydration. Invest Ophthalmol Vis Sci.

[22] Cınar Y., Cingü A.K., Türkcü F.M., Çınar T., Yüksel H., Özkurt Z.G., Çaça I. (2014). Comparison of accelerated and conventional corneal collagen cross-linking for progressive keratoconus.. Cutan. Ocul. Toxicol..

[23] Jain V., Gazali Z., Bidayi R. (2014). Isotonic riboflavin and HPMC with accelerated cross-linking protocol.. Cornea.

[24] Hafezi F., Mrochen M., Iseli H.P., Seiler T. (2009). Collagen crosslinking with ultraviolet-A and hypoosmolar riboflavin solution in thin corneas.. J. Cataract Refract. Surg..

[25] Raiskup F., Spoerl E. (2011). Corneal cross-linking with hypo-osmolar riboflavin solution in thin keratoconic corneas.. Am. J. Ophthalmol..

[26] Koppen C., Wouters K., Mathysen D., Rozema J., Tassignon M.J. (2012). Refractive and topographic results of benzalkonium chloride-assisted transepithelial crosslinking.. J. Cataract Refract. Surg..

[27] Raiskup F., Pinelli R., Spoerl E. (2012). Riboflavin osmolar modification for transepithelial corneal cross-linking.. Curr. Eye Res..

[28] Raiskup F., Veliká V., Veselá M., Spörl E. (2015). Cross-Linking in Keratoconus: “Epi-off” or “Epi-on”?.. Klin. Monatsbl. Augenheilkd..

[29] Lesniak S.P., Hersh P.S. (2014). Transepithelial corneal collagen crosslinking for keratoconus: six-month results.. J. Cataract Refract. Surg..

[30] Soeters N., Wisse R.P., Godefrooij D.A., Imhof S.M., Tahzib N.G. (2015). Transepithelial versus epithelium-off corneal cross-linking for the treatment of progressive keratoconus: a randomized controlled trial.. Am. J. Ophthalmol..

[31] Kocak I., Aydin A., Kaya F., Koc H. (2014). Comparison of transepithelial corneal collagen crosslinking with epithelium-off crosslinking in progressive keratoconus.. J. Fr. Ophtalmol..

[32] Filippello M., Stagni E., O’Brart D. (2012). Transepithelial corneal collagen crosslinking: bilateral study.. J. Cataract Refract. Surg..

[33] Wollensak G., Iomdina E. (2009). Biomechanical and histological changes after corneal crosslinking with and without epithelial debridement.. J. Cataract Refract. Surg..

[34] Daxer A., Mahmoud H.A., Venkateswaran R.S. (2010). Corneal crosslinking and visual rehabilitation in keratoconus in one session without epithelial debridement: new technique.. Cornea.

[35] Coskunseven E., Jankov M.R., Hafezi F., Atun S., Arslan E., Kymionis G.D. (2009). Effect of treatment sequence in combined intrastromal corneal rings and corneal collagen crosslinking for keratoconus.. J. Cataract Refract. Surg..

[36] Daugimont L., Baron N., Vandermeulen G., Pavselj N., Miklavcic D., Jullien M.C., Cabodevila G., Mir L.M., Préat V. (2010). Hollow microneedle arrays for intradermal drug delivery and DNA electroporation.. J. Membr. Biol..

[37] Mastropasqua L., Lanzini M., Curcio C., Calienno R., Mastropasqua R., Colasante M., Mastropasqua A., Nubile M. (2014). Structural modifications and tissue response after standard epi-off and iontophoretic corneal crosslinking with different irradiation procedures.. Invest. Ophthalmol. Vis. Sci..

[38] Bikbova G., Bikbov M. (2014). Transepithelial corneal collagen cross-linking by iontophoresis of riboflavin.. Acta Ophthalmol..

[39] Gaudana R., Ananthula H.K., Parenky A., Mitra A.K. (2010). Ocular drug delivery.. AAPS J..

[40] Yasukawa T., Ogura Y., Tabata Y., Kimura H., Wiedemann P., Honda Y. (2004). Drug delivery systems for vitreoretinal diseases.. Prog. Retin. Eye Res..

[41] Bunsen R., Roscoe H.E. (1855). Photochemische untersuchungen.. poggendorffs annalen.

[42] Wernli J., Schumacher S., Spoerl E., Mrochen M. (2013). The efficacy of corneal cross-linking shows a sudden decrease with very high intensity UV light and short treatment time.. Invest. Ophthalmol. Vis. Sci..

[43] Kanellopoulos A.J. (2012). Long term results of a prospective randomized bilateral eye comparison trial of higher fluence, shorter duration ultraviolet A radiation, and riboflavin collagen cross linking for progressive keratoconus.. Clin. Ophthalmol..

[44] Gatzioufas Z., Richoz O., Brugnoli E., Hafezi F. (2013). Safety profile of high-fluence corneal collagen cross-linking for progressive keratoconus: preliminary results from a prospective cohort study.. J. Refract. Surg..

[45] Richoz O., Tabibian D., Hammer A., Majo F., Nicolas M., Hafezi F. (2014). The effect of standard and high-fluence corneal cross-linking (CXL) on cornea and limbus.. Invest. Ophthalmol. Vis. Sci..

[46] Beshtawi I.M., Akhtar R., Hillarby M.C., O’Donnell C., Zhao X., Brahma A., Carley F., Derby B., Radhakrishnan H. (2013). Biomechanical properties of human corneas following low- and high-intensity collagen cross-linking determined with scanning acoustic microscopy.. Invest. Ophthalmol. Vis. Sci..

[47] Beshtawi I.M., Akhtar R., Hillarby M.C., O’Donnell C., Zhao X., Brahma A., Carley F., Derby B., Radhakrishnan H. (2015). Biomechanical Changes of Collagen Cross-Linking on Human Keratoconic Corneas Using Scanning Acoustic Microscopy.. Curr. Eye Res..

[48] Kamaev P., Friedman M.D., Sherr E., Muller D. (2012). Photochemical kinetics of corneal cross-linking with riboflavin.. Invest. Ophthalmol. Vis. Sci..

[49] Hammer A., Richoz O., Arba Mosquera S., Tabibian D., Hoogewoud F., Hafezi F. (2014). Corneal biomechanical properties at different corneal cross-linking (CXL) irradiances.. Invest. Ophthalmol. Vis. Sci..

[50] Herekar S.V.

[51] Mazzotta C, Traversi C, Paradiso AL, Latronico ME, Rechichi M (2014). Pulsed Light Accelerated Crosslinking versus Continuous Light Accelerated Crosslinking.. One-Year Results. J Ophthalmol.

[52] Mazzotta C., Traversi C., Caragiuli S., Rechichi M. (2014). Pulsed vs continuous light accelerated corneal collagen crosslinking: *in vivo* qualitative investigation by confocal microscopy and corneal OCT.. Eye.

[53] Kanellopoulos A.J., Asimellis G. (2014). Keratoconus management: long-term stability of topography-guided normalization combined with high-fluence CXL stabilization (the Athens Protocol).. J. Refract. Surg..

[54] Kanellopoulos A.J., Asimellis G. (2015). Novel placido-derived topography-guided excimer corneal normalization with cyclorotation adjustment: enhanced athens protocol for keratoconus.. J. Refract. Surg..

[55] Kymionis G.D., Grentzelos M.A., Kankariya V.P., Liakopoulos D.A., Karavitaki A.E., Portaliou D.M., Tsoulnaras K.I., Pallikaris I.G. (2014). Long-term results of combined transepithelial phototherapeutic keratectomy and corneal collagen crosslinking for keratoconus: Cretan protocol.. J. Cataract Refract. Surg..

[56] Kymionis G.D., Grentzelos M.A., Liakopoulos D.A., Paraskevopoulos T.A., Klados N.E., Tsoulnaras K.I., Kankariya V.P., Pallikaris I.G. (2014). Long-term follow-up of corneal collagen cross-linking for keratoconus the Cretan study.. Cornea.

[57] Said D.G., Elalfy M.S., Gatzioufas Z., El-Zakzouk E.S., Hassan M.A., Saif M.Y., Zaki A.A., Dua H.S., Hafezi F. (2014). Collagen cross-linking with photoactivated riboflavin (PACK-CXL) for the treatment of advanced infectious keratitis with corneal melting.. Ophthalmology.

[58] Price M.O., Tenkman L.R., Schrier A., Fairchild K.M., Trokel S.L., Price F.W. (2012). Photoactivated riboflavin treatment of infectious keratitis using collagen cross-linking technology.. J. Refract. Surg..

[59] Papaioannou L., Miligkos M., Papathanassiou M. (2016). Corneal collagen cross-linking for infectious keratitis: A systematic review and meta-analysis.. Cornea.

